# Asthma incidence, remission, relapse and persistence: a population-based study in southern Taiwan

**DOI:** 10.1186/s12931-014-0135-9

**Published:** 2014-11-12

**Authors:** Tsung-Ju Wu, Chang-Fu Wu, Yungling Leo Lee, Tzuen-Ren Hsiue, Yue Leon Guo

**Affiliations:** Institute of Occupational Medicine and Industrial Hygiene, National Taiwan University, Taipei, Taiwan; Division of Chest Medicine, Department of Internal Medicine, Kaohsiung Municipal Min-Sheng Hospital, Kaohsiung, Taiwan; Institute of Epidemiology and Preventive Medicine, National Taiwan University, Taipei, Taiwan; Division of Chest Medicine, Department of Internal Medicine, National Cheng Kung University Hospital, Medical College, National Cheng Kung University, Tainan, Taiwan; Department of Environmental and Occupational Medicine, National Taiwan University (NTU) College of Medicine and NTU Hospital, Taipei, Taiwan

**Keywords:** Adult asthma, Asthma, Adulthood, Asthma incidence, Asthma remission, Asthma relapse, Asthma persistence, Atopic disease, Puberty

## Abstract

**Background:**

In western countries, late-onset asthmatics are more severe than early-onset asthmatics in clinic-based studies. However, whether asthma occurrence rates were higher in late ages than in younger ages was inconclusive. This information is essentially lacking in Asian population.

**Methods:**

The participants were schoolchildren’s parents recruited from 94 elementary and middle schools in 2004. A cross-sectional self-administered questionnaire was sent through the children to their parents to survey their respiratory health. We investigated typical asthma symptoms occurring at different ages and subsequent remission or relapse after the first asthma event. Person-years of the participants from birth to the time of survey were used as the denominator.

**Results:**

Among the 25,377 participants consisting of 949,807 total person-years, 860 reported ever having asthma. Highest incidences occurred at ages 0–12 and 36–40 years. The incidence of asthma was higher in males before puberty, and higher in females after puberty, with overall incidences 1.00 and 0.77 per 1000 person-years for females and males, respectively. Participants with late-onset asthma (onset age >12 years) comprised a large portion of adult current asthmatics. More than 52% of persistence or relapse was observed in early-onset asthma (onset age ≤12 years). The younger birth cohort had a more prominent later peak of asthma incidence than the older one.

**Conclusions:**

In Asian population, asthma occurrence showed a U-shape age distribution with a prominent second peak in the thirties. A high proportion of early-onset asthma relapsed and most of late-onset asthma persisted or relapsed in adulthood.

## Background

Asthma is a heterogeneous disease with a wide variety of phenotypes. Among different asthma phenotype categories, the phenotypes defined by age at onset in an early to late spectrum are most natural and can differentiate clinical and pathophysiological difference. In clinical studies, onset age of asthma can differentiate atopic status [1,2], severity of airway obstruction [3] or lung function loss [1], medication needs and frequency of healthcare utilization [2].

Nevertheless, age-specific incidence of asthma was widely variable among some large-scale surveys of western countries [4-9]. These studies have varied in their investigated population, environmental backgrounds and study time periods. As the prevalence of asthma is known to be higher in western countries than in eastern countries [10], there is probable difference in age-specific incidence of asthma between western and eastern countries.

The natural course of asthma is also heterogeneous. The possible courses of asthma activity after its onset can include persistence, complete remission or interspersion of remission and relapse. Both relapse and persistent wheezing are related to lung function decline indicated by severity of airway obstruction [11]. Some epidemiological studies have focused on remission of asthma [6,12,13]. These studies showed that the proportion of remission was higher among early-onset than late-onset asthmatics. However, few epidemiological studies focused on the relapse of asthma [14,15], which were limited by relatively small sample sizes and shorter follow-up periods. A large-scale study to investigate asthma relapse is needed.

Estimation of incidence and understanding of the natural course of asthma onset at different ages and subsequent outcomes could benefit investigation for disease burden, potential risk factors and targeted management plan. In 2004, we conducted a population-based study in southern Taiwan to investigate age-specific incidence of asthma and natural course of asthma, in terms of remission, relapse and persistence [16].

## Methods

### Study design

**Figure 1:**
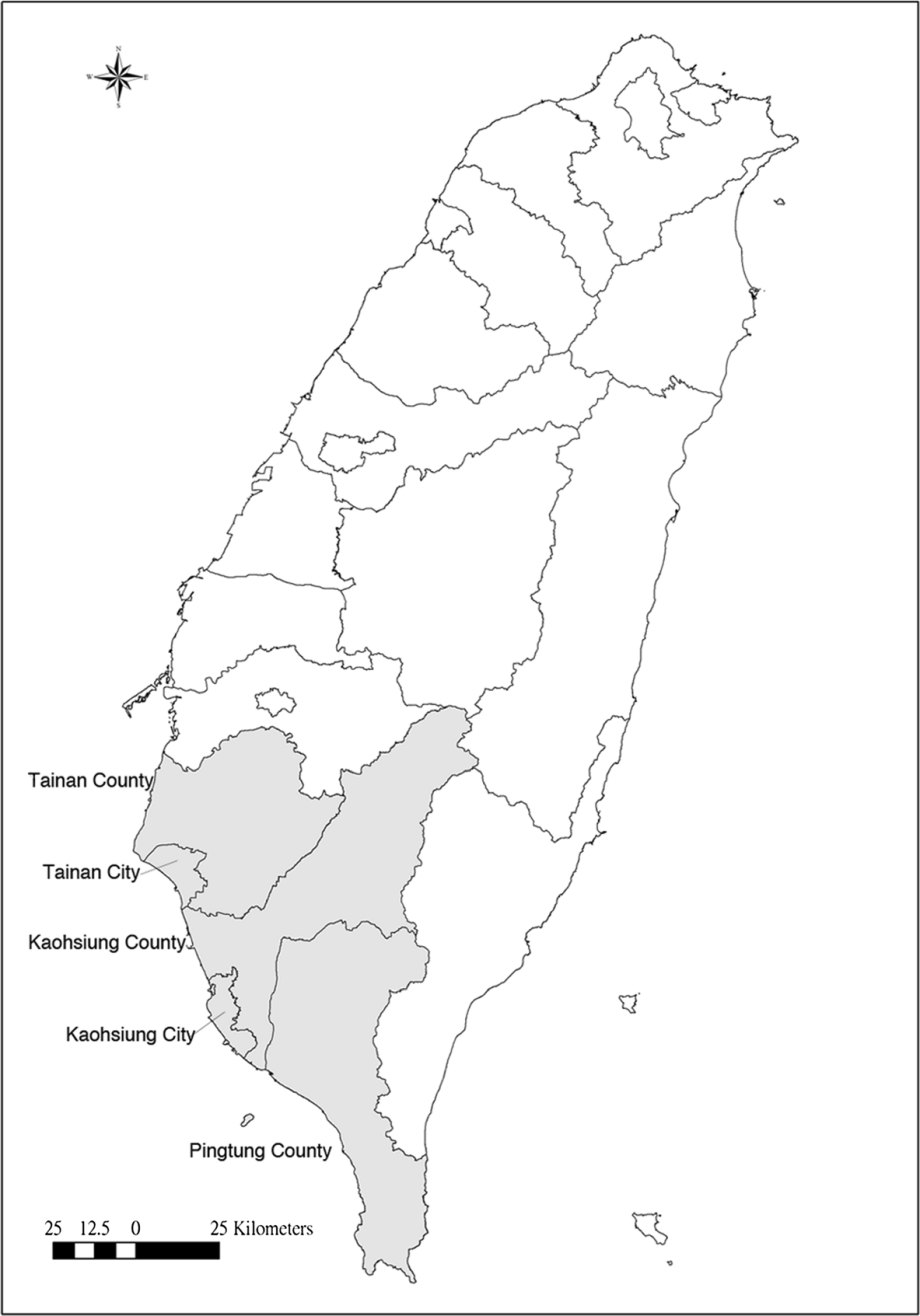
**Five administrative districts of the study in southern Taiwan in 2004.**

The southernmost part of Taiwan Island (southern Taiwan) was comprised of five administrative districts in 2004 - Tainan City, Tainan County, Kaohsiung City, Kaohsiung County, and Pintung County (Figure 1). The population and territory of this area in 2002 were 5,501,747 people and 7,914 square kilometers. In 2004, we conducted a school-based cross-sectional study including retrospective survey of respiratory diseases and symptoms for schoolchildren and their parents in southern Taiwan. Twenty of the 189 middle schools and 74 of the 627 elementary schools were randomly selected for investigation. In each school, random sampling of candidate participants from each grade stratification was conducted. This study focused on the parents’ respiratory health. The questionnaire was taken home and answered by one of the parents. The related health information was collected with a questionnaire, which was a Chinese version modified from the questionnaire of the American Thoracic Society and the Division of Lung Diseases (ATS-DLD-78) [17,18]. The revised questionnaire was evaluated and verified by a pulmonologist and two epidemiologists for appropriate translation to Mandarin. The study protocol was approved by the Institutional Review Board (Human Study Committee) at the National Cheng Kung University Hospital. It complied with the Declaration of Helsinki [19]. Each participant provided informed consent for the study.

### Asthma related definitions

Before the core questionnaire, typical asthma-like symptoms, for example wheezing in the chest, nocturnal dyspnea, and night cough unassociated with a cold, were delineated. The definition of asthma was a positive response to the question, “Have you ever experienced the aforementioned asthma-like symptoms?” If the subject fulfilled the definition of having asthma, he/she would be further asked, “At what age did they start?”

Asthma remission was defined as no asthma symptoms for more than 3 years from the first asthma event to the time of survey. If an asthmatic had any 3 years of symptom-free period, he/she would be further asked, “After 3 years of symptom-free period or longer, have these symptoms recurred?”, and “At what age did the asthma symptoms recurred?”

Complete remission was defined as asthmatics who had been symptom-free for more than three years without relapse and without asthma medicine in the last year when answering the questionnaire. Relapsed asthmatics were those who had remission for more than 3 years with subsequent relapse or asthma medication use in the last year when answering the questionnaire. Persistent asthma was defined as not being symptom-free for any three year period of time.

Prevalences of potential risk factors for asthma incidence, including active smoking, passive smoking, maternal asthma, paternal asthma, maternal allergic rhinitis, paternal allergic rhinitis, maternal atopic eczema and paternal atopic eczema, were investigated among different birth cohorts.

### Statistical analysis

Incidence rates were the number of participants with newly onset asthma divided by the total person-years at risk. Person-year was defined as the time period in which a subject was at risk for developing asthma. The estimates of asthma incidence, adjusted for potential confounding factors, were analyzed by Poisson modeling. An interaction term between ‘birth cohort’ and ‘age at onset’ was used to test whether incidence by age at onset varies according to the birth cohort. Cochran-Armitage trend test was used to evaluate trends of prevalence of potential risk factors among different birth cohorts. Stata 11.0 software (StataCorp LP, College Station, Texas) was used for analyses.

## Results

### Characteristics of study subjects

**Figure 2:**
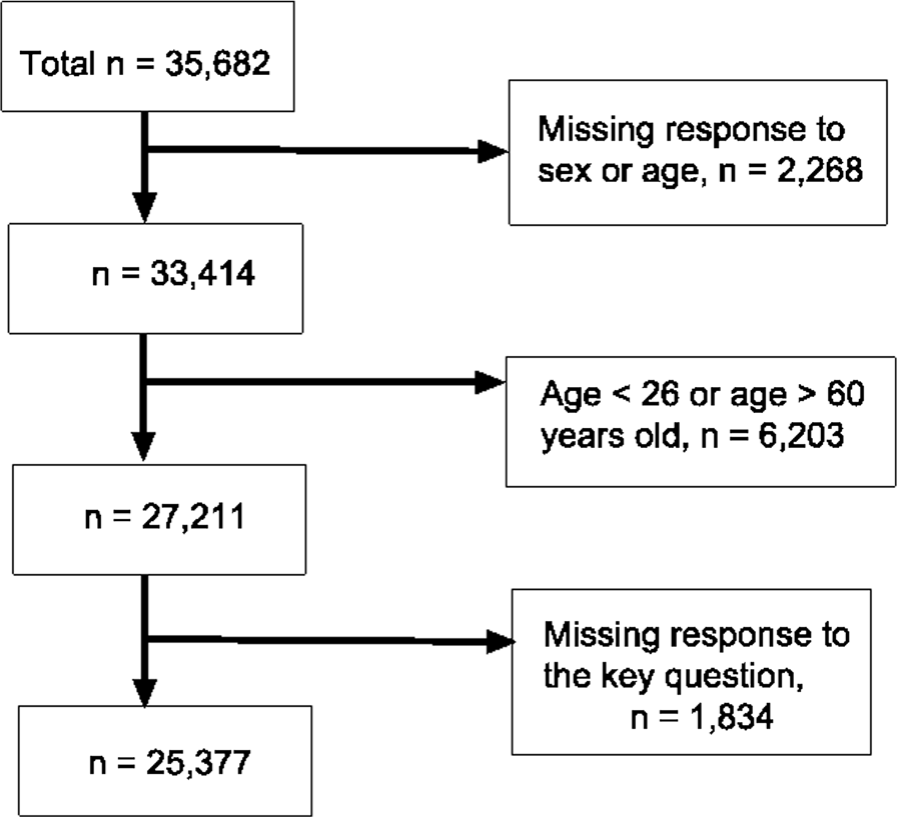
**The flow chart describing the enrollment of the study population.**

We surveyed the parents of 35,682 children from 94 elementary and middle schools. A total of 10,305 subjects were excluded because of inadequate demographic information, being older than 60 years of age or missing responses to the key question (Figure 2). Among those with age and sex data, the distributions of age and sex were not statistically different between those included in the final analysis and those who excluded. Data from 25,377 subjects were satisfactory, with a response rate of 71.1%. Among these participants, 886 cases of asthma were identified. Due to potential confounding with chronic obstructive pulmonary disease and heart failure among those who answered with an onset age of 41 years or older, the analysis was carried out only to the age of 40.

### Incidence

Among the 25,377 participants and their 949,807 total person-years from birth to the time of survey, 860 reported ever having asthma. The crude incidence was higher in females than in males. Highest incidences occurred at ages 0 to 12 years and 36 to 40 years. The age-specific incidences ranged from 0.45/1000 person-years in age group 19–25 years to 2.03/1000 person-years in age group 36–40 years. The overall asthma incidences show an increasing trend with a later year of birth (Table 1).

**Table 1:** **Numbers of asthmatics, person-years and incidence of asthma by onset age, birth cohort and sex**

	**No. of cases/No. at risk**	**Person-years**	**Incidence (95% C.I.)/1000 person-years**
Whole sample	860/25,377	949,807	0.91 (0.85 ~ 0.97)
Onset age (years)			
0-6	161/25,377	177,639	0.91 (0.78 ~ 1.06)
7-12	151/25,377	152,262	0.99 (0.85 ~ 1.16)
13-15	50/25,377	76,131	0.66 (0.50 ~ 0.87)
16-18	39/25,377	76,131	0.51 (0.37 ~ 0.70)
19-25	80/25,377	177,639	0.45 (0.36 ~ 0.56)
26-30	103/24,767	123,835	0.83 (0.69 ~ 1.01)
31-35	152/21,006	105,030	1.45 (1.23 ~ 1.70)
36-40	124/12,228	61,140	2.03 (1.70 ~ 2.42)
Birth cohort (age in years)			
1978-1969 (26–35)	220/5,771	182,851	1.20 (1.05 ~ 1.37)
1968-1964 (36–40)	349/9,371	347,321	1.01 (0.91 ~ 1.12)
1963-1959 (41–45)	201/7,273	298,193	0.67 (0.59 ~ 0.77)
1958-1948 (46–56)	90/2,962	121,442	0.74 (0.60 ~ 0.91)
Sex			
Male	300/10,016	387,436	0.77 (0.69 ~ 0.87)
Female	560/15,361	562,371	1.00 (0.92 ~ 1.08)

**Figure 3:**
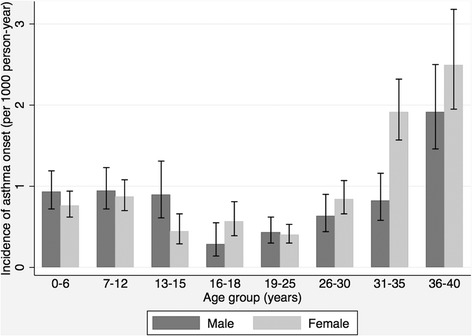
**Incidence rates of asthma onset in men and women by age group.** Rates were adjusted for birth cohort by using Poisson regression model.

The age-specific incidences were adjusted for the birth cohort by using Poisson regression. The bimodal onset age pattern remained; however, the later peak became more prominent. The males tended to have asthma before the age of 15 years; in contrast, the females tended to have asthma after the age of 16 years (Figure 3).

### Complete remission, relapse and persistence

**Figure 4:**
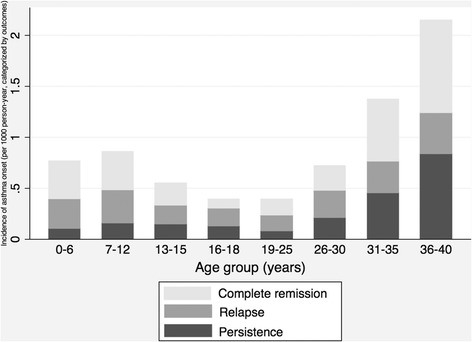
**Incidence of asthma onset by age group, categorized by current outcomes, namely, complete remission, relapse and persistence.** Rates were adjusted for birth cohort and sex by using Poisson regression model. Among 860 cases, 29 of them missed the responses to asthma outcomes.

The proportions of complete remission were similar among different onset age groups. The highest proportion of persistence occurred at the onset age group of 36–40 years. In contrast, the highest proportion of relapse occurred at the onset age group of 16–18 years (Table 2). In sum, participants with later onset of asthma tended to have active asthma in adulthood; among those with active asthma, a similar bimodal pattern of onset was observed (Figure 4).

**Table 2:** **Proportions of current asthma outcomes for each onset age group**

**Onset age (years)**	**No. of cases/No. at risk (%)**	**Complete remission**	**Remission followed by relapse**	**Persistence**
0-6	161/25,377 (0.63%)	49%	38%	14%
7-12	151/25,377 (0.60%)	44%	38%	18%
13-15	50/25,377 (0.20%)	39%	33%	27%
16-18	39/25,377 (0.15%)	23%	44%	33%
19-25	80/25,377 (0.32%)	40%	40%	20%
26-30	103/24,767 (0.42%)	34%	37%	29%
31-35	152/21,006 (0.72%)	44%	23%	33%
36-40	124/12,228 (1.01%)	42%	19%	39%

**Figure 5:**
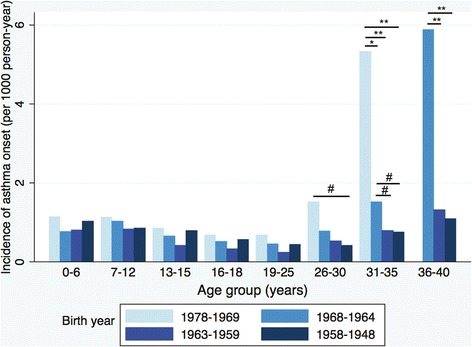
**Incidence of asthma onset by age group, in different birth cohorts.** The incidence rate ratios were statistically different between the latter birth cohorts and the earlier birth cohorts in age group 26–30, 31–35 and 36–40, respectively, through the Poisson regression model. Asthmatics in the birth cohort born 1978 to1969 did not reach the ages from 36 to 40 years when the study was undergoing. **P <0.001; *P <0.01; ^#^P <0.05.

The bimodal incidence pattern was repeatedly observed among different birth cohorts of this study and a more prominent later peak was observed among the younger birth cohort (Figure 5).

Only the prevalence of passive smoking showed a significant increasing trend from the older to the younger birth cohorts (Table 3).

**Table 3:** **Prevalence rates (%) of potential risk factors for asthma onset among different birth cohorts**

	**Birth cohort**				**P** ^*^
	**1978-1969**	**1968-1964**	**1963-1959**	**1958-1948**	
Active smoking	20.12	23.32	28.31	30.71	0.15
Passive smoking	51.89	43.18	39.58	35.93	<0.001
Paternal asthma	1.94	2.34	2.79	2.53	0.23
Paternal allergic rhinitis	6.79	5.75	5.72	4.59	0.22
Paternal atopic eczema	1.39	1.01	1.14	0.95	0.22
Maternal asthma	1.98	2.07	2.19	2.53	0.81
Maternal allergic rhinitis	5.84	5.65	5.18	3.61	0.08
Maternal atopic eczema	1.07	0.88	0.78	0.51	0.81

^*^Cochran-Armitage trend test.

## Discussion

To our knowledge, this is one of the largest cross-sectional studies which included natural history of asthma incidence, remission and relapse equivalent to a long follow-up period and with total person-years amounting to 949,807. The study included a wide range of ages from birth to adulthood, which would have hardly been accomplished by a longitudinal study. Moreover, we observed that the incidence of asthma featured a U-shaped age distribution with a prominent second peak at ages 30–40 years and late-onset asthmatics (onset age >12 years) comprised a large portion of adult current asthmatics.

A U-shape age-specific incidence pattern of asthma was reported in a large cross-sectional study in Italy [6]. Compared with our study, the study time period was similar; however, the overall incidence of asthma was higher in Italy than in Taiwan. In our study, the high incidences of asthma in individuals of higher ages were more prominent than those in Italy. In the United Kingdom, a cohort study following subjects up to age of 33 years showed a similar increasing trend in asthma incidence after puberty [5]. However, there were differences among the three studies. Our study and the study in Italy were cross-sectional studies with retrospective components. The study in UK was a prospective study with longitudinal follow-up. This current study used ATS-DLD questionnaire, but the other two studies did not. However, the definition of asthma was similar in the three studies, and was based on typical asthma symptoms and wheezing in the chest. Overall, the main findings from these three studies were rather similar both in the U-shape incidence of asthma onset, and in increasing secular trend.

In contrast, in North America, three studies of different time periods, i.e. 1964–1983 in Rochester, 1996–2007 in Ontario, and 2006–2008 in the US, did not find the elevated asthma incidence after age of 25 years [4,8,9]. Similar findings in two different genetic backgrounds (Taiwan and Italy), and divergent findings in similar genetic backgrounds (UK, Canada and US) pointed to differential environmental factors for adulthood asthma. As non-atopy is preponderant in late-onset asthma, a non-atopic mechanism such as air pollution or microbial toxin [20,21] may contribute to the increased incidence of late-onset asthma.

Due to limited space of the questionnaire, thorough history of past respiratory diseases was not obtained in this study. However, effects of respiratory infectious diseases on risk of asthma were observed clearly in childhood, and to a less extent after adolescence [5,22]; thus past respiratory infectious diseases might have limited influence on the results of our study. On the other hand, parental history of allergic disease has effect both on early and late-onset asthma [23,24]. However, we found no significant trend for the prevalence of hereditary factors among birth cohorts in this current study (Table 3), indicating that parental allergic disease was not the factor for our observation of increased prevalence in asthma.

The U-shape incidence pattern was repeatedly observed among different birth cohorts which implied that bimodal onset pattern was not caused by recall bias. The late-onset peak of incidence seemed to be more prominent in the later birth cohorts (Figure 5). Regarding effect of smoking on asthma onset, both active smoking and passive smoking are known related to late-onset asthma, but only passive smoking is related to early-onset asthma [5]. This current study found decreasing prevalence of active smoking but increasing passive smoking (Table 3). These seemingly conflicting results could have been caused by actual dropping in smoking, and the participants’ higher alert towards passive smoking in the more recent birth cohort. As a result, we believe smoking did not play important role in increasing secular trend of asthma. The hereditary risk factors and environmental risk factors both increased the risks of late-onset asthma [16]. However, the prominent increasing second peak in the later birth cohort was too rapid to be accounted for by changes in gene frequencies. Thus, environmental factors might play a more important role on this phenomenon. As air pollution [25] and phthalates exposure [26] were associated with late-onset asthma and showed an increasing trend, these factors warranted further studies.

An alternative explanation of the high incidence in the age 31–40 years is that the result is driven by the better remembrance of asthma incidence among age 36–45 years compared to older subjects in this study. Although possibilities of better recall of asthma occurrence in age 31–40 years among the youngest participants as compared to older participants cannot be totally ruled out, we did not expect a large enough effect as to distort the observed second peak, since the oldest participants were only aged 56 years, younger than the common onset age of dementia.

Late-onset asthma comprised a large portion of active asthma cases in adulthood. Moreover, the later the birth cohort (e.g., 1978–1969 in this study), the higher the later peak appeared. This implies that late-onset asthmatics may contribute to an even larger portion of active asthmatics in future adulthood. As late-onset asthma is reported to be more severe than early-onset asthma in several clinic-based studies, and more severe asthma is associated with higher economic costs [27], late-onset asthma may carry a greater economic impact in the future.

Previous longitudinal studies, which followed their participants to young adulthood, showed that a third of early-onset asthmatics had continuing symptoms of asthma, including persistence and relapse [11,28]. However, in this current study we found a higher proportion of persistence or relapse (more than 52%) in the group of asthmatics with onset age before 12 years. The difference might have been caused by the difference in the ages at the last follow-up, i.e., 26 [11], 30 [28], and 38 years as median age of last contact with the participants in the two longitudinal studies and this current study, respectively. Therefore, the relapse rate of early-onset asthma could have been underestimated in these two longitudinal studies [11,28].

In our study, the incidence of asthma was higher in males before puberty, but higher in females after puberty till age 40 years, which was consistent with two studies in western countries [4,6]. As a result, a higher lifetime incidence of asthma was noted in females, which was consistent with the findings in western countries as well [6,29].

Using the same questionnaire (ATS-DLD) to assess the prevalence of childhood asthma (0–12 years), a study in Japan [30] showed a higher proportion of asthma onset at age 0–6 years (79.5-84.9%) than age 7–12 years. In contrast, this study found similar rates of asthma onset in these two age groups. However, both studies found higher incidence among boys than girls in the childhood. On the other hand, Nishima et al. showed the higher prevalence of childhood asthma in the younger birth cohort, which is compatible with a higher second peak of incidence observed in the later birth cohort in our study.

Previous studies showed that the prevalence of allergic sensitization is higher in prepubescent boys than girls [31]. In addition, in prepubescent ages, boys have larger lungs than girls for the same height [32] and greater specific airway resistance [33]. These findings give a plausible explanation that airway hyperresponsiveness is higher in prepubescent boys than girls [34].

However, the sex difference pattern changes after puberty. In adolescence, both growth of airways relative to lung volumes and growth in height relative to lung function are faster in males than females [35,36]. This gives a plausible explanation that females are more likely to have airway hyperresponsiveness after ages 8–12 years [37]. Hormonal effects on the airways were also frequently associated with the sex difference in asthma incidence, which was supported by the association of changes of exhaled nitric oxide and hormones during the menstrual cycle [38].

In summary, the sex difference of asthma incidence before and after puberty may be caused by changes in airway hyperresponsiveness, allergic sensitization, the pattern of growth in lung function, pubertal hormone changes during adolescence, etc. However, the causes of prominent later peak of asthma incidence in southern Taiwan and Italy have not been thoroughly studied. Age-related lung function loss, environmental factors, etc. may contribute to this phenomenon.

This current study has several strengths. In adult asthma studies, our study was one of the largest studies with a rather large number of person-years of follow-up. The study population was randomly selected from urban, suburban and rural areas, which increased its representativeness from a wide variety of environmental backgrounds. We especially included an inquiry for relapse, which could disclose the real natural history of asthma and allow for distinguishing from pathophysiological different conditions like persistent asthma.

### Several limitations of the study

The recall of the asthma events - the onset, remission, relapse and persistence - could have been subject to recall bias. The absolute incidence rates could have been unreliable prior to age 6 years and the information was possibly acquired from their parents. A previous longitudinal study showed that 59% of asthma-like symptoms before age 3 years disappeared in later life, which is regarded as transient wheeze; thus the absolute incidence rates could have been overestimated [39]. On the other hand, incidence rates could have been underestimated because of the “telescoping effect” – a tendency to attribute the onset closer to the time of the questionnaire survey [40]. However, comparing to previous survey of asthma in Taiwan, comparable lifetime prevalences were found. In a 1967 birth cohort, lifetime prevalence of asthma at age 7 years was reported by parents to be 2.1% [41]; whereas the corresponding figure was 2.8% in this study (data not shown).

For the purpose of achieving a better response rate, we did not assign which of the parents to answer the questionnaire. This could have led to an overestimate of asthma’s incidence in the population, as the parent with asthma symptoms was more likely to complete the questionnaire than their spouse. However, comparing to previous survey of asthma in Taiwan, comparable lifetime prevalences were found. In a 1981 birth cohort, lifetime prevalence was reported to be 9.1% [42]; whereas the corresponding figure was 9.5% in this study (data not shown). Therefore, such overestimation might have existed but was small.

We did not perform a test to see if subjects could accurately place other events in their personal histories. In our study, the incidence of asthma was defined as the occurrence of a typical asthma attack, which is a crucial event and is less likely to be subject to recall bias. Similarly, in a retrospective study in Italy, a question for the age of the first asthma event has good reproducibility over a 10-year time period in subjects of comparable age with our study [6].

While we categorized asthma outcome (remission, relapse or persistence) in each onset age group, fewer years of observation for late-onset asthma could have led to the highest proportion of persistence occurring at the onset age of 36–40 years. However, taking the highest incidence rate and sum of relapse and persistence rates together, more active asthmatics were still observed in this age group. Thus we would not have misclassified the asthma activity among different onset age groups in the population.

The perception of complete remission, relapse or persistence of asthma could be attributed to adherence to asthma medications and treatment regimens. However, we have evidence to show otherwise, that late-onset asthmatics actually received more asthma medications (short-acting β2-agonist and inhaled corticosteroid) and more healthcare use (data not shown), which implied that more active cases in late-onset asthma were not due to poor adherence to medications.

As the study sample was school-based, we only surveyed the parents of children aged 6–15 years. One problem is that we were unable to include those without children in this survey. However, asthma was related to delayed time to pregnancy, but not contribute to “childless” [43]. In this current study, the reported numbers of children were not different between asthmatic and non-asthmatic parents (data not shown). Therefore, we did not expect this to distort our overall findings.

As our study population included a wide range of ages, there was the possibility that asthma diagnoses and treatments would have different effects on different generations. We further examined the ratios of physician-diagnosed asthma and typical asthma symptoms and found that there was no secular trend of the ratios among different birth cohorts (60%, 55%, 57%, 50% from earlier to latter birth cohorts, respectively). Furthermore, we used typical asthma symptoms as the outcome measure, which could lessen the impact of improved diagnoses within different generations. The asthmatics in younger generations might have better treatments, which could impact the prevalence of asthma in different generations. However, in our study, late-onset asthmatics received more medications but had greater disease activity; thus the main conclusions – bimodal onset of asthma and more active late-onset asthmatics - would not be altered.

The criteria of asthma were based on reported typical asthma symptoms, but without clinical confirmation. Therefore, our findings regarding asthma should be viewed as fulfilling epidemiological criteria, but not necessarily clinical criteria of asthma. Nevertheless, the questionnaire used in this study was based on ATS-DLD-78 questions on asthma and asthma-like symptoms in adults, which have been validated [44] and has been widely used. Use of this questionnaire allows for comparisons with other epidemiological investigations.

## Conclusions

In a cross-sectional survey of people born 1948 to 1978 in southern Taiwan, we found a noteworthy portion of late-onset asthma, as well as a higher relapse rate of early-onset asthma than longitudinal studies conducted in Europe and New Zealand. Asthmatics in the later-onset age groups comprised a large portion of active asthma cases in adulthood. Incidence of asthma was higher in males before puberty, but in females after puberty, resulting in a higher lifetime incidence among females. These findings provided useful information to the understanding of asthma in later years of life. Knowledge has been relatively lacking on the risk factors for late-onset asthma, and the importance for further investigations in this area cannot be overemphasized.

## Abbreviation

ATS-DLD: American Thoracic Society and the Division of Lung Diseases
